# Supplementary material for: RNA-Protein Interactome at the Hepatitis E Virus Internal Ribosome Entry Site
Source: Microbiol Spectr. 2023 Jun 29;11(4):e02827-22. doi: 10.1128/spectrum.02827-22 (PMC10434006; doi:10.1128/spectrum.02827-22)
Supplement: Supplemental file 1 — Supplemental material. Download spectrum.02827-22-s0001.pdf, PDF file, 1.4 MB [file spectrum.02827-22-s0001.pdf]

Fig S1

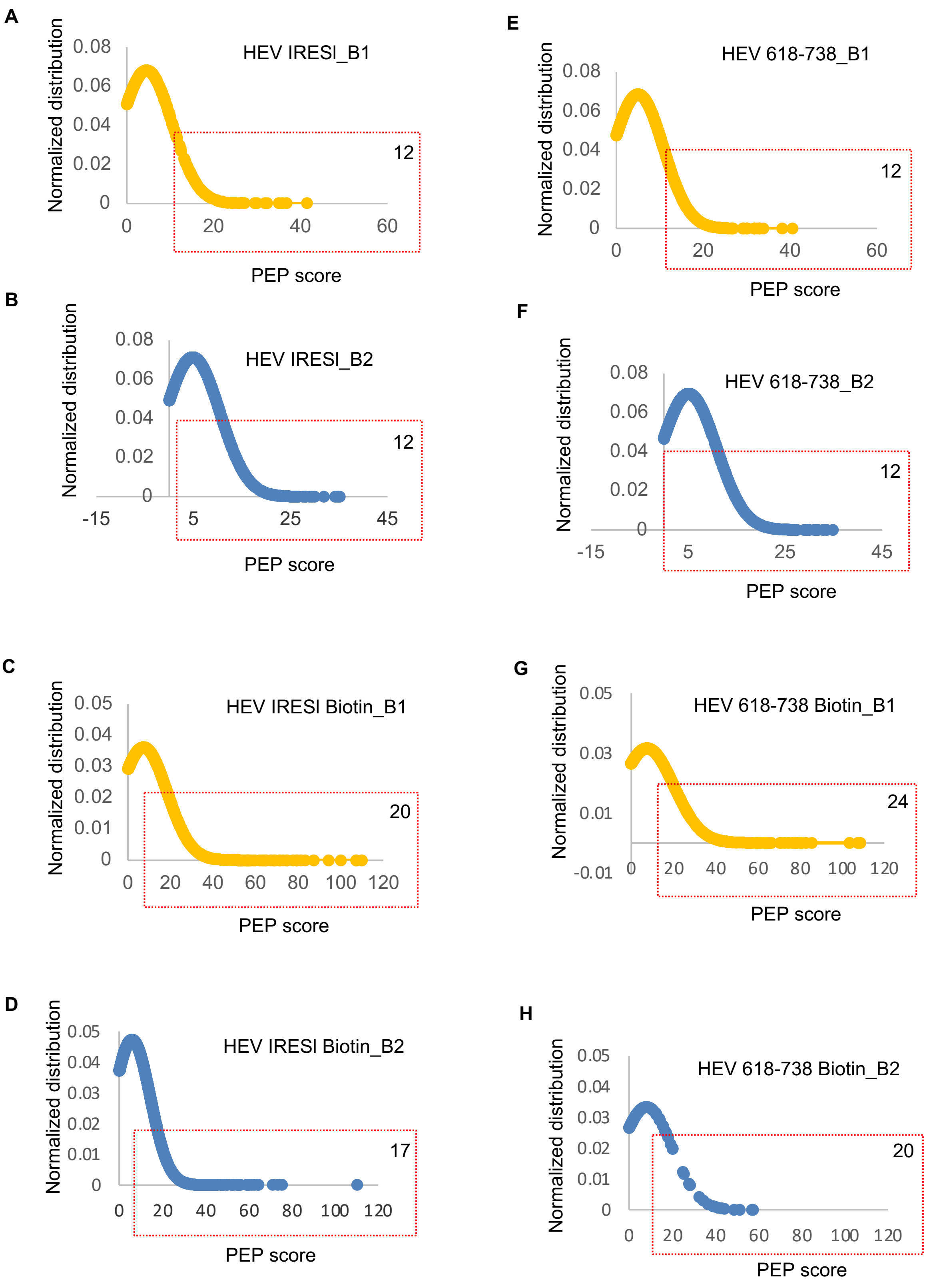
A
HEV IRESI_B1
Normalized distribution
0.08
0.06
0.04
0.02
0
0
20
40
60
12
PEP score
B
HEV IRESI_B2
Normalized distribution
0.08
0.06
0.04
0.02
0
-15
5
25
45
12
PEP score
C
HEV IRESI Biotin_B1
Normalized distribution
0.05
0.04
0.03
0.02
0.01
0
0
20
40
60
80
100
120
20
PEP score
D
HEV IRESI Biotin_B2
Normalized distribution
0.05
0.04
0.03
0.02
0.01
0
0
20
40
60
80
100
120
17
PEP score
E
HEV 618-738_B1
Normalized distribution
0.08
0.06
0.04
0.02
0
0
20
40
60
12
PEP score
F
HEV 618-738_B2
Normalized distribution
0.08
0.06
0.04
0.02
0
-15
5
25
45
12
PEP score
G
HEV 618-738 Biotin_B1
Normalized distribution
0.05
0.03
0.01
-0.01
0
20
40
60
80
100
120
24
PEP score
H
HEV 618-738 Biotin_B2
Normalized distribution
0.05
0.04
0.03
0.02
0.01
0
0
20
40
60
80
100
120
20
PEP score

Fig S2

A

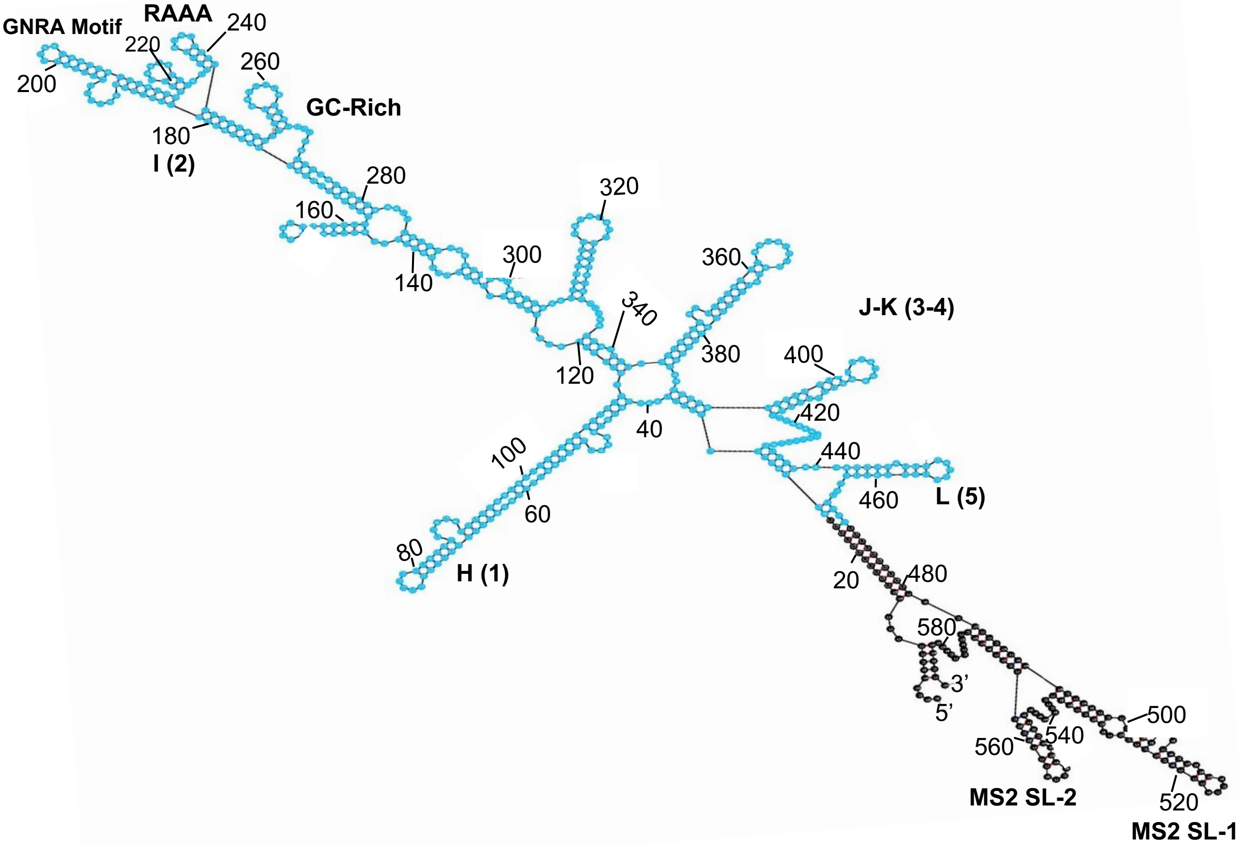
GNRA Motif
RAAA
GC-Rich
I (2)
J-K (3-4)
L (5)
H (1)
MS2 SL-2
MS2 SL-1
200
220
240
260
180
280
160
320
300
140
360
340
120
380
400
420
40
100
440
460
60
80
20
480
580
3'
5'
500
540
560
520

B

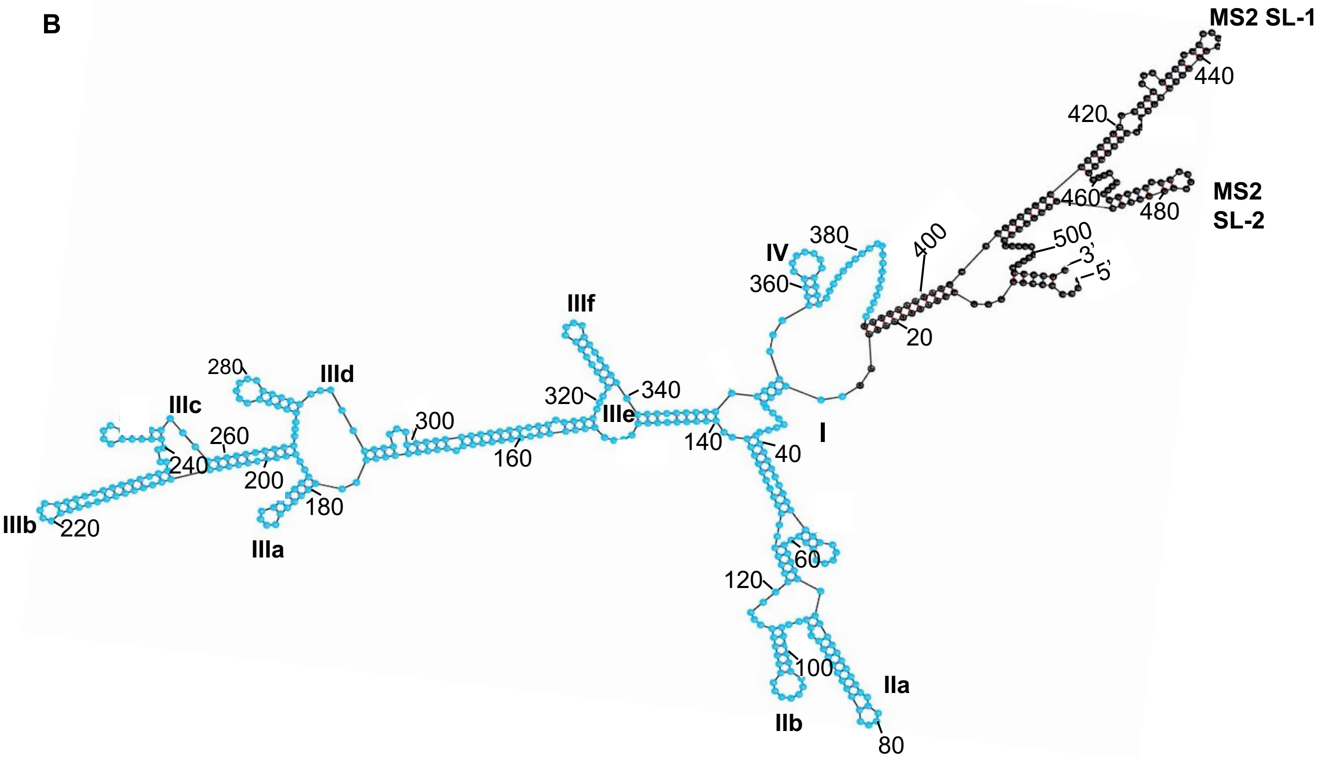
MS2 SL-1
440
420
MS2
SL-2
460
480
380
400
500
3'
5'
IV
360
20
IIIf
280
IIId
320
340
IIIc
300
IIIe
260
I
240
160
140
40
200
180
IIIb
220
IIIa
60
120
100
IIa
IIb
80

Fig S3

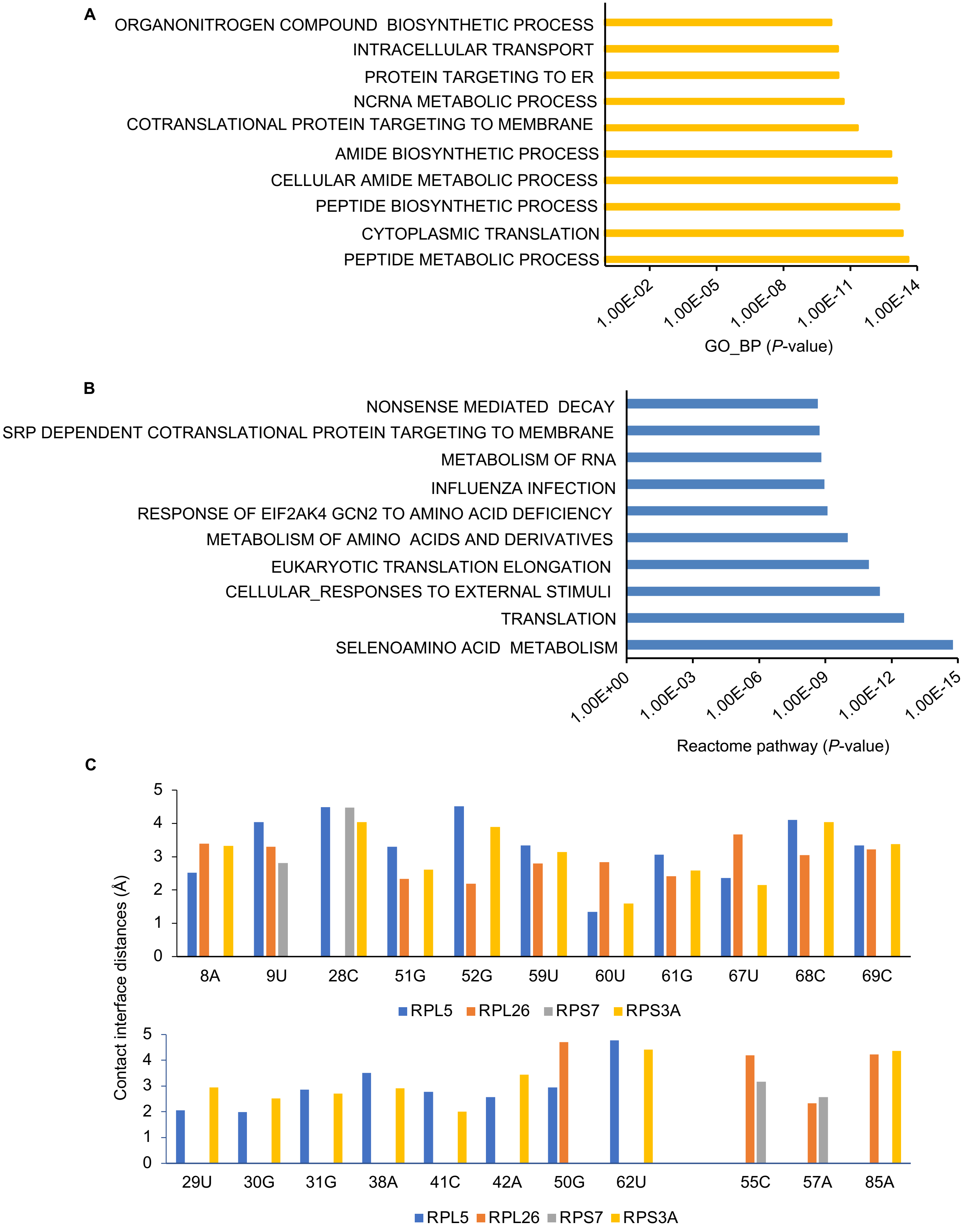
A
ORGANONITROGEN COMPOUND BIOSYNTHETIC PROCESS
INTRACELLULAR TRANSPORT
PROTEIN TARGETING TO ER
NCRNA METABOLIC PROCESS
COTRANSLATIONAL PROTEIN TARGETING TO MEMBRANE
AMIDE BIOSYNTHETIC PROCESS
CELLULAR AMIDE METABOLIC PROCESS
PEPTIDE BIOSYNTHETIC PROCESS
CYTOPLASMIC TRANSLATION
PEPTIDE METABOLIC PROCESS
1.00E-02
1.00E-05
1.00E-08
1.00E-11
1.00E-14
GO_BP (P-value)
B
NONSENSE MEDIATED DECAY
SRP DEPENDENT COTRANSLATIONAL PROTEIN TARGETING TO MEMBRANE
METABOLISM OF RNA
INFLUENZA INFECTION
RESPONSE OF EIF2AK4 GCN2 TO AMINO ACID DEFICIENCY
METABOLISM OF AMINO ACIDS AND DERIVATIVES
EUKARYOTIC TRANSLATION ELONGATION
CELLULAR_RESPONSES TO EXTERNAL STIMULI
TRANSLATION
SELENOAMINO ACID METABOLISM
1.00E+00
1.00E-03
1.00E-06
1.00E-09
1.00E-12
1.00E-15
Reactome pathway (P-value)
C
Contact interface distances (Å)
0
1
2
3
4
5
8A
9U
28C
51G
52G
59U
60U
61G
67U
68C
69C
RPL5
RPL26
RPS7
RPS3A
29U
30G
31G
38A
41C
42A
50G
62U
55C
57A
85A
RPL5
RPL26
RPS7
RPS3A

Fig S4

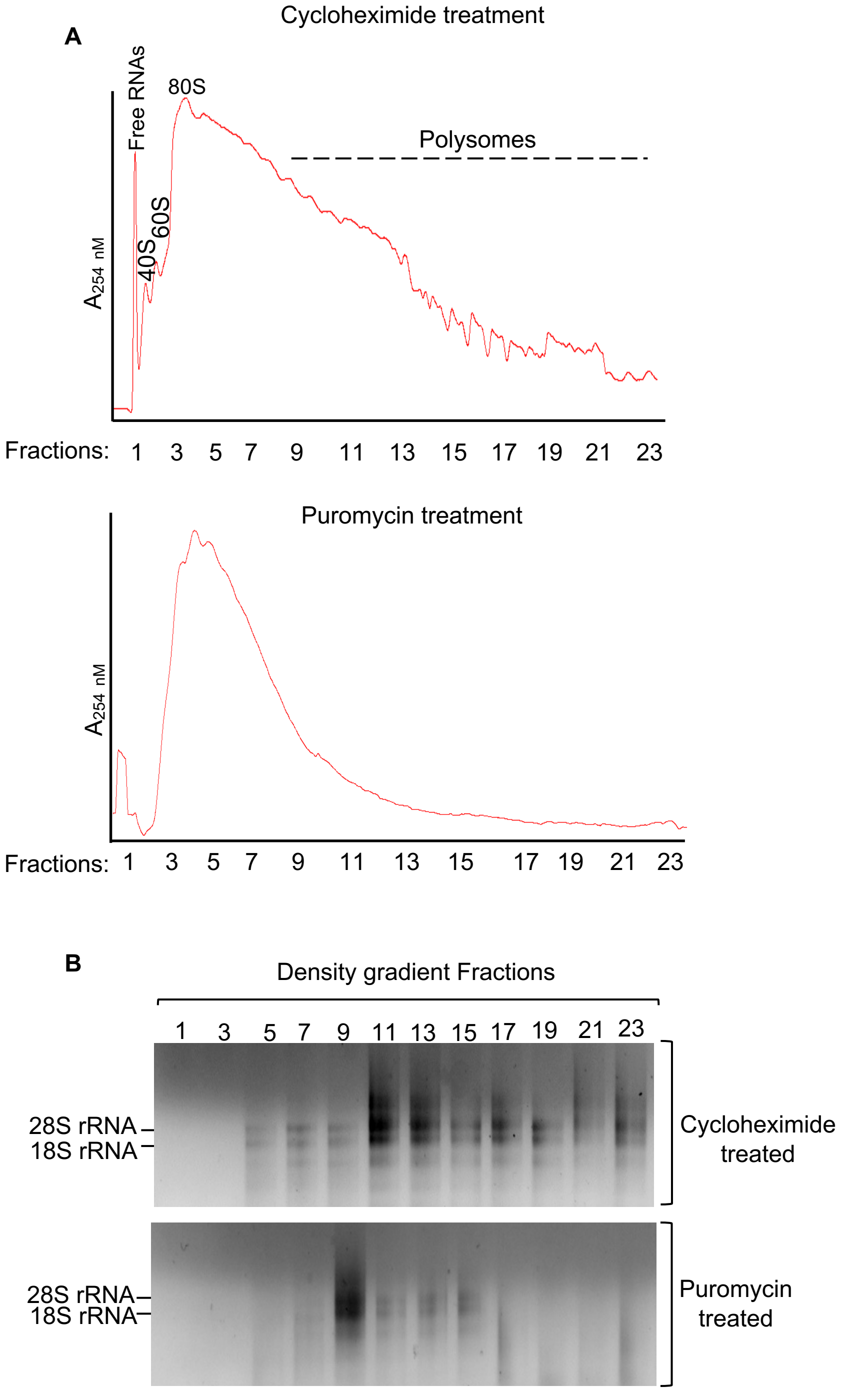
A
Cycloheximide treatment
Free RNAs
80S
40S
60S
Polysomes
A254 nM
Fractions: 1 3 5 7 9 11 13 15 17 19 21 23
Puromycin treatment
A254 nM
Fractions: 1 3 5 7 9 11 13 15 17 19 21 23
B
Density gradient Fractions
1 3 5 7 9 11 13 15 17 19 21 23
28S rRNA
18S rRNA
Cycloheximide treated
28S rRNA
18S rRNA
Puromycin treated

Supplementary Figure Legends

Figure S1. Normalized distribution plots of the peptides identified by LC-MS/MS.

(A-H) Normalized distribution plots showing all peptides identified in LC-MS/MS against their respective PEP scores. B1 and B2 denote biological replicates of the same samples. PEP score range considered for protein identification is shown by a dotted-lined box.

Figure S2. Predicted secondary structure of the FMDV and HCV IRES RNA.

(A) Predicted secondary structure of the FMDV IRES RNA (shown in blue) fused to the MS2-coat protein-binding RNA. MS2 SL-1 and MS2 SL-2 represent MS2 coat proteinbinding RNA motifs. Five SLs of the IRES are numbered H-L (1-5) and span positions 26113, 114-344, 343-418 and 417- 470, respectively. GNRA, RAAA, GC-rich motifs are as indicated. (B) Predicted secondary structure of the HCV IRES RNA (shown in blue) fused to the MS2-RNA. MS2 SL-1 and MS2 SL-2 represent MS2 coat protein-binding RNA motifs. Four stem-loops (SL) of the IRES are numbered as I, II, III (a-e) and IV.

Figure S3. Bioinformatics analysis of the HEV IRESl RNA-host protein interaction.

(A) Graphical representation of the top 10 Biological processes (sorted by P-values) enriched in the HEV IRESl RNA-protein interactome, analyzed by the GSEA tool (B) Graphical representation of the top 10 Reactome pathways (sorted by P-values) enriched in the HEV IRESl RNA-protein interactome, analyzed by the GSEA tool. (C) Quantitative pair-wise contact interface distance calculation between the HEV IRESl RNA and the indicated ribosomal proteins. The pair-wise interface residues contacts (measured in the form of distance in Å) was calculated for each complex. The common contact nucleotides of the HEV IRESl RNA are shown in the upper and lower panels, respectively.

Figure S4. Ribosome fractionation profiles of cycloheximide or puromycin treated Huh7 cells.

(A) HEK 293T cells were treated with cycloheximide for 30 min, followed by preparation of the cytosolic extract and ultracentrifugation on 10-50% sucrose gradients. Equal volume fractions were collected from the top in an automated fraction collector. Real-time profile of the RNA content in each fraction was measured at A_{254nm} and the plot generated by the application software is shown. Peaks corresponding to free mRNAs, 40S, 60S, 80S ribosome and polysome fractions are indicated based on earlier reports (upper panel). HEK 293T cells were treated with puromycin

for 30 min, followed by preparation of the cytosolic extract and ultracentrifugation on 10-50% sucrose gradients. Equal volume fractions were collected from the top in an automated fraction collector. Real-time profile of the RNA content in each fraction was measured at A_{254nm} and the plot generated by the application software is shown (lower panel). (B) Formaldehyde-agarose gel electrophoresis of total RNA isolated from the sucrose density gradient fractions of the Huh7 cells, cotransfected with the pSuper-control RNA plasmid, pSuper-HEV IRESI plasmid and pRL-TK plasmid and treated with cycloheximide (upper panel) or puromycin (lower panel).

Table S1. List of the HEV IRESl RNA interaction partners, identified by RaPID-LC-MS-MS

Protein name	prot_desc	prot_score	No. of unique peptide	pep_score
EF2	Elongation factor 2	1568	6	59.63
TBA3C	Tubulin alpha-3C/D chain	929	1	24.2
SYTC	Threonyl-tRNA synthetase, cytoplasmic	285	5	17.17
ROA2	Heterogeneous nuclear ribonucleoproteins A2/B1	285	2	35.14
RL7A	60S ribomal protein L7a	262	3	19
BA2L2	Protein BAT2-like 2	75	1	74.55
NUFP2	Nuclear fragile X mental retardation-interacting protein 2	75	1	46.91
SYVC	Valyl-tRNA synthetase	56	8	18.02
DHX9	ATP-dependent RNA helicase A	53	7	42.62
SYTC2	Probable threonyl-tRNA synthetase 2, cytoplasmic	53	5	17.17
ARP19	cAMP-regulated phphoprotein 19	53	1	52.79
ENSA	Alpha-endulfine	53	1	52.79
RL24	60S ribomal protein L24	53	1	35.28
HS105	Heat shock protein 105 kDa	50	6	20.75
CATA	Catalase	49	2	49.14
DDX10	Probable ATP-dependent RNA helicase DDX10	42	9	42.18
LARP4	La-related protein 4	42	1	41.97
MIMIT	Mimitin, mitochondrial	41	1	41.09
DBNL	Drebrin-like protein	39	1	36.56
LBR	Lamin-B receptor	38	1	37.78
AIMP1	Aminoacyl tRNA synthetase complex-interacting multifunctional protein 1	37	3	20.08
NSF	Vesicle-fusing ATPase	37	1	19
VW5B1	von Willebrand factor A domain-containing protein 5B1	35	5	40.75
CEA16	Carcinoembryonic antigen-related cell adhesion molecule 16	35	1	34.88

CCD65	Coiled-coil domain-containing protein 65	34	5	33.67
CN043	Uncharacterized protein C14orf43	34	4	33.93
PUM1	Pumilio homolog 1	34	2	34.22
TXLNG	Gamma-taxilin	33	10	33.34
RFA1	Replication protein A 70 kDa DNA-binding subunit	33	4	32.54
ARSJ	Arylsulfatase J	33	1	32.32
CCD67	Coiled-coil domain-containing protein 67	32	8	32.5
BIVM	Basic immunoglobulin-like variable motif-containing protein	32	3	32.47
YTHD2	YTH domain family protein 2	32	2	32.45
RSBNL	Round spermatid basic protein 1-like protein	32	1	32.09
SPD2A	SH3 and PX domain-containing protein 2A	32	1	31.88
UBAP2	Ubiquitin-associated protein 2	32	1	32.35
GLSK	Glutaminase kidney isoform, mitochondrial	31	1	30.81
MRE11	Double-strand break repair protein MRE11A	31	1	31.09
PRAF3	PRA1 family protein 3	31	1	20.25
CENPP	Centromere protein P	30	1	29.73
ERP29	Endoplasmic reticulum resident protein 29	30	1	23.18
LASP1	LIM and SH3 domain protein 1	30	1	29.73
TM59L	Transmembrane protein 59-like	30	1	29.73
COL10	Collectin-10	29	3	29.32
ZYX	Zyxin	29	2	29.49
GATS	Putative protein GATS	29	1	29.38
PPN	Papilin	29	1	28.77
SC31A	Protein transport protein Sec31A	29	1	29.47
SYQ	Glutaminyl-tRNA synthetase	29	1	29.38
PAPS2	Bifunctional 3~-phphoadenine 5~-phphulfate synthase 2	28	3	27.81
K0649	Protein KIAA0649	28	2	27.59
NUP88	Nuclear pore complex protein Nup88	28	2	25.32
NALDL	N-acetylated-alpha-linked acidic dipeptidase-like protein	28	1	27.97
VPS39	Vam6/Vps39-like protein	28	1	28.29

ZC12C	Probable ribonuclease ZC3H12C	28	1	27.67
LRRF2	Leucine-rich repeat flightless-interacting protein 2	27	5	27.36
M4K4	Mitogen-activated protein kinase kinase kinase kinase 4	27	5	19.92
F195A	Protein FAM195A	27	2	26.9
MDN1	Midasin	26	2	17.57
ACRBP	Acrin-binding protein	26	1	26.38
UBP7	Ubiquitin carboxyl-terminal hydrolase 7	26	1	26.35
GRM7	Metabotropic glutamate receptor 7	25	3	25.19
CHMP5	Charged multivesicular body protein 5	25	1	25.45
MGAP	MAX gene-associated protein	24	13	21.31
ATP7A	Copper-transporting ATPase 1	24	6	24.32
TRM1	N(2),N(2)-dimethylguanine tRNA methyltransferase	24	4	24.25
CND3	Condensin complex subunit 3	24	1	24.31
K2C74	Keratin, type II cytkeletal 74	24	1	23.82
QTRD1	Queuine tRNA-ribyltransferase subunit QTRTD1	24	1	24.1
S28A3	Solute carrier family 28 member 3	24	1	23.51
ZN469	Zinc finger protein 469	24	1	24.29
DYRK3	Dual specificity tyrine-phphorylation-regulated kinase 3	23	1	23.42
EZH1	Histone-lysine N-methyltransferase EZH1	23	1	23.02
EZH2	Histone-lysine N-methyltransferase EZH2	23	1	23.02
FA81A	Protein FAM81A	23	1	22.56
MMP12	Macrophage metalloelastase	23	1	23.36
FA98A	Protein FAM98A	22	5	22.05
XPO4	Exportin-4	22	3	20.68
TEX15	Testis-expressed sequence 15 protein	22	2	21.88
CF050	Uncharacterized protein C6orf50	22	1	22.14
NIN	Ninein	22	1	22.37
VP37A	Vacuolar protein sorting-associated protein 37A	22	1	21.61
CB067	Uncharacterized protein C2orf67	21	10	21.12
EDRF1	Erythroid differentiation-related factor 1	21	8	19.38
YE031	Transmembrane protein ENSP00000382582	21	5	21.06

TT21A	Tetratricopeptide repeat protein 21A	21	4	17.03
ABCG2	ATP-binding cassette sub-family G member 2	21	2	21.29
NMD3B	Glutamate [NMDA] receptor subunit 3B	21	2	20.88
TEX28	Testis-specific protein TEX28	21	2	20.57
ABHD6	Monoacylglycerol lipase ABHD6	21	1	21.27
ACBG2	Long-chain-fatty-acid--CoA ligase ACSBG2	21	1	20.91
COLQ	Acetylcholinesterase collagenic tail peptide	21	1	21.16
GMFB	Glia maturation factor beta	21	1	21.34
NIT1	Nitrilase homolog 1	21	1	21.38
S26A7	Anion exchange transporter	21	1	20.95
YE015	Ankyrin repeat and death domain-containing protein	21	1	24.63
ZN236	Zinc finger protein 236	21	1	20.51
AGAP2	Arf-GAP with GTPase, ANK repeat and PH domain-containing protein 2	20	4	20.09
SYLC	Leucyl-tRNA synthetase, cytoplasmic	20	4	18.18
DC1I1	Cytoplasmic dynein 1 intermediate chain 1	20	2	19.91
HPHL1	Hephaestin-like protein 1	20	2	20.04
MYPC2	Myin-binding protein C, fast-type	20	1	20.25
PRAF2	PRA1 family protein 2	20	1	20.25
CO4A1	Collagen alpha-1(IV) chain	19	9	19.05
PGS2	Decorin	19	5	19.27
RASA1	Ras GTPase-activating protein 1	19	5	19.15
RBL1	Retinoblastoma-like protein 1	19	5	19.69
CO033	Uncharacterized protein C15orf33	19	4	22.99
DYH9	Dynein heavy chain 9, axonemal	18	18	18.01
LKAP	Limkain-b1	18	14	17.68
TRPM3	Transient receptor potential cation channel subfamily M member 3	18	9	22.95
AKT1	RAC-alpha serine/threonine-protein kinase	18	1	17.93
CCD34	Coiled-coil domain-containing protein 34	16	1	20.12
CNN1	Calponin-1	16	1	20.87
KNTC1	Kinetochore-associated protein 1	15	8	18.24

Table S2. Summary of human fetal liver library screening by the Y3H assay to identify the host factors that interact with the HEV IRESl element

Description	No. of Colonies
Number of clones screened	4 x 10^6
Number of colonies on (LUH$^-$+10mM 3AT)	395
Number of colonies containing human liver cDNA insert	285
Number of colonies containing unique cDNA insert	75
Unique protein coding sequences in frame with the AD	8
Number of positive clones upon retransformation of the p3HR2-HEV IRESl and the isolated liver library clone	8

Table S3. Analysis of interaction of the HEV IRESI RNA-interacting host proteins (found in Y3H library screening) with the FMDV and HCV IRES RNA[a]

Name of the host protein	HEV IRESI	FMDV IRES	HCV IRES
GREBP	+++	+	+
PPIG	+	-	+++
RPL5	+++	-	+
RPL26	++	-	+++
RPL41	+++	-	-
RPS3A	+++	+++	+++
RPS7	+++	-	+
RPS15A	+++	-	-

[a] +++ : Strong Interaction; + : weak interaction; - : No interaction
